# Stem cell therapy – Hype or hope? A review

**DOI:** 10.4103/0972-0707.58329

**Published:** 2009

**Authors:** Roopa R Nadig

**Affiliations:** Department of Conservative Dentistry and Endodontics, Dayananda Sagar College of Dental Sciences, Bangalore, Karnataka, India

**Keywords:** DPSC, regeneration, scaffolds, stem cell

## Abstract

While the regeneration of a lost tissue is known to mankind for several years, it is only in the recent past that research on regenerative medicine/dentistry has gained momentum and eluded the dramatic yet scientific advancements in the field of molecular biology. The growing understanding of biological concepts in the regeneration of oral/dental tissues coupled with experiments on stem cells is likely to result in a paradigm shift in the therapeutic armamentarium of dental and oral diseases culminating in an intense search for “biological solutions to biological problems.” Stem cells have been successfully isolated from variety of human tissues including orofacial tissues. Initial evidence from pioneering studies has documented the likely breakthrough that stem cells offer for various life-threatening diseases that have so far defeated modern medical care. The evidence gathered so far has propelled many elegant studies exploring the role of stem cells and their manifold dental applications. This review takes you on a sojourn of the origin of stem cells, their properties, characteristics, current research, and their potential applications. It also focuses on the various challenges and barriers that we have to surmount before translating laboratory results to successful clinical applications heralding the dawn of regenerative dentistry.

## INTRODUCTION

The sojourn of science has unraveled and understood that the secret of life lies in the “DNA,” thanks to Sir James Watson and Crick for their epoch making a historic discovery. In our endeavor to demystify the DNA, we have realized that scientific discoveries in cellular, developmental, and molecular biology have truly revolutionized our collective understanding of the biological processes that could greatly impact and dramatically change our lives in the future.

In the new millennium, where biology and biotechnology have replaced chemistry, we are exploring “biological solutions to biological problems.” Owing to the extraordinary advances taking place in the field of cellular and molecular biology, we are on the verge of a paradigm shift, evolving from offering simple mechanical care to consider biological solutions to health promotion, risk assessment, diagnosis, treatment, and even prognosis.

Although stem cell technology is just emerging, the regeneration of body parts is hardly a new concept. The regenerative capability of a living creature was recorded as early as 330 BC, when Aristotle observed that a lizard could grow back the lost tip of its tail. Since then, there have been slow but steady attempts at understanding the regenerative capabilities of human being and it is only in the last decade that we have seen an information explosion in the area of stem cell research. Stem cells are likely to revolutionize the entire health care delivery. The time is certainly ripe for all of us to familiarize ourselves with the following: what are stem cells, their characteristics, their potential applications, current research translating to therapy, and possible barriers of its application from the bench to the bedside/chair.

## WHAT ARE STEM CELLS AND THEIR CHARACTERISTICS?

They are unspecialized cells with an extraordinary ability to self-renew, capable of differentiating into one or more specialized cell types playing a crucial role in homeostasis and tissue repair.

When called into action following an injury, a stem cell self-renews – undergoes cell division and gives rise to one daughter stem cell and one progenitor cell. A progenitor cell is an intermediate cell type formed before it achieves a fully differentiated state. It is regarded as committed to differentiating along a particular cellular developmental pathway of stem cells:

Stem cell → Stem cell + Progenitor cell → Differentiated cell

Based on their origin, stem cells are categorized either as embryonic stem cells (ESCs) or as postnatal stem cells/somatic stem cells/adult stem cells (ASCs).

### Characteristics

1. Totipotency: generate all types of cells including germ cells (ESCs).
2. Pluripotency: generate all types of cells except cells of the embryonic membrane.
3. Multipotency: differentiate into more than one mature cell (MSC).
4. Self-renewal: divide without differentiation and create everlasting supply.
5. Plasticity: MSCs have plasticity and can undergo differentiation. The trigger for plasticity is stress or tissue injury which upregulates the stem cells and releases chemoattractants and growth factors.

Among the types of differentiation are:

1. Direct differentiation: a specific type of cell in a special niche developed in a multistep unidirectional pathway (e.g., MSCs differentiating into osteoblasts/fibroblasts).
2. Transdifferentiation: direct conversion of one cell type to another different cell type (e.g., blood cells into brain cells and vice versa).
3. Dedifferentiation: a unipotent stem cell becoming a multipotent one.
4. Cell fusion: a stem cell fusing with a somatic cell resulting in another lineage (e.g., ESCs fuse *in vitro* with HSCs and neuronal cells).

### Embryonic stem cells

Embryonic stem cells (ESCs) are derived from embryos that are 2–11 days old called blastocysts. They are best grown from supernumerary embryos obtained from *in vitro* fertilization centers. They are totipotent – cells virtually capable of differentiating into any type of cell including the germ cell. ESCs are considered immortal as they can be propagated and maintained in an undifferentiated state indefinitely. These stem cells have the highest potential to regenerate and repair diseased tissue and organs in the body.[1,2] However, the therapeutic benefit of ESCs is bogged by a controversy owing to the belief that the process of extraction of stem cells from an embryo destroys the embryo itself and some view this as taking life, thereby, raising moral and ethical concerns. Further, it is difficult to control the growth and differentiation of the embryonic stem cell posing risk of tumorogenicity and teratoma formation. While research is on to overcome some of these shortfalls as of now, ESCs are not so far used therapeutically and have only remained an excellent platform for research.

### Adult stem cells

Adult stem cells are found in most adult tissues. They are multipotent – capable of differentiating into more than one cell type but not all cell types.[3] The plasticity of an adult stem cell is described as its ability to expand beyond its potential irrespective of the parent cell from which it is derived. For example, dental pulp stem cells not only develop into tooth tissue but also have the ability to differentiate into neuronal tissue.

Depending on their origin, adult stem cells can be further classified as hemopoetic stem cells (HSCs) and mesenchymal stem cells (MSCs). HSCs are obtained either from cord blood or peripheral blood. MSCs are those that originate from the mesoderm layer of the fetus and in the adult reside in a variety of tissues such as the bone marrow stem cells (BMSCc), limbal stem cells, hepatic stem cells, dermal stem cells, etc.

Stem cells have also been isolated from orofacial tissues which include adult tooth pulp tissue, pulp tissue of deciduous teeth, periodontal ligament, apical papilla, and buccal mucosa. Gronthos *et al*. have isolated stem cells from adult human dental pulp (DPSCs) that exhibit a similar immunophenotype to bone marrow stem cells.[4] Stem cells from human exfoliated deciduous teeth (SHED) represent a unique population of multipotent stem cells that are easily accessible and are more immature in the cell hierarchy than the adult pulp stem cells.[5] Using a similar methodology, multipotent stem cells from the human periodontal ligament (PDLCs) have also been described.[6] Recently, a new population of mesenchymal stem cells (MSCs) residing in the apical papilla of incompletely developed teeth (SCAP) have been isolated and demonstrated in elegant studies.[7,8]

## WHY STEM CELL RESEARCH?

Advances in stem cell research have explored many therapeutic avenues and the insight gained by a pilot proof of concept studies promises a plethora of unimaginable benefits:

- They are useful in functional genomic studies to understand human embryonic gene expression, genomic data mining, and bioinformatics.[9]
- They serve to study biological processes which help in understanding human developmental disorders like birth defects, cancers, etc.
- They are new means of creating human disease models for drug discovery and development. It serves as an alternative to animal toxicology thereby hastening the drug to the market.[10]
- The most crucial and exciting of all stem cell uses is cell-based therapy.

### Cell-based therapy

Adult stem cells have been used in pilot studies as potential cell-based therapy for various diseases. The following stem cell characteristics make them good candidates for cell-based therapy:[9]

1. Potential to be harvested from patients
2. High capacity of cell proliferation in culture
3. Ease of manipulation to replace existing nonfunctional genes via gene splicing methods
4. Ability to migrate to host target tissues (homing)
5. Ability to integrate into host tissues and interact with the surrounding tissues.

### Process of cell-based therapy

Stem cells derived from either peripheral blood, cord blood, bone marrow, or any adult tissue transported in the right medium to the laboratory is centrifuged, trypsinized, and propagated under ideal conditions and stored in the master cell bank (MCB). The MCB is further passaged to yield colonies of stem cells, given the right inductive signals using appropriate growth factors to allow them to differentiate into required cell types. These are injected or implanted into a patient as cell-based therapy. Homing will ensure that the stem cells reach the site of injury/tissues.

Today, the two common methods of cell delivery are intravenous injection (direct delivery of cells) and cell encapsulation systems (indirect delivery of cells using a carrier). The cell encapsulation approach uses a biocompatible, biodegradable material construct that is seeded with cells and implanted into defects in order to regenerate the lost tissue.[11]

## POTENTIAL APPLICATIONS IN MEDICINE

Stem cells are being explored for a variety of chronic debilitating diseases that have so far escaped remedial measures from traditional allopathic approaches with a hope that cell therapy would repair, repopulate, replace, and rewire tissues and organs regenerating hope and kindling confidence in such therapies.

Because of the overwhelming success of animal studies, numerous clinical trials are now going on world over.[12–16] Various therapeutic programs in either pilot or proof of concept studies are exploring the role of cell replacement therapy under conditions like Parkinson's disease, spinal cord injury, heart failure, hematological disease, cancer, arthritis, diabetes, and peripheral vascular disease. Interim results from the pilot studies are encouraging and have led the US FDA to permit Phase III clinical trials for acute and steroid refractory graft versus host disease and also Crohn's disease, Phase II clinical trials for the repair of heart tissue following a heart attack, the protection of pancreatic islet cells in patients with type 1 diabetes, and the repair of lung tissue in patients with chronic obstructive pulmonary disease. An injectable formulation of mesenchymal stem cells, for arthritis in the knee, is also being evaluated (data on file – www.osiristx.com). Even in India, we are not far behind since Phase II proof of concept studies has been approved and is underway (http://clinicaltrisals.gov/).

## POTENTIAL APPLICATIONS IN DENTISTRY

The regenerative potential of adult stem cells obtained from various sources including dental tissues has been of interest for clinicians over the past years and most research is directed toward achieving the following:

- Regeneration of damaged coronal dentin and pulp
- Regeneration of resorbed root, cervical or apical dentin, and repair perforations
- Periodontal regeneration
- Repair and replacement of bone in craniofacial defects
- Whole tooth regeneration.

### Regeneration of damaged coronal dentin and pulp

To this date, no restorative material has been able to mimic all physical and mechanical properties of tooth tissue. Furthermore, we have not been successful in providing an ideal solution to certain situations, such as an immature tooth with extensive coronal destruction and reversible pulpitis. If the regeneration of tooth tissue is possible in these situations, it facilitates physiologic dentin deposition that forms an integral part of the tooth thereby restoring structural integrity, minimizing interfacial failure, microleakage, and other consequent complications. Similarly, young permanent teeth that require apexogenesis or apexification are the perfect candidates for the regeneration of pulp as they allow completion of both vertical and lateral root development, improving the long-term prognosis. However, pulp regeneration in fully formed teeth may not be of great benefit, although there is sufficient evidence to say that a restored vital tooth serves longer than a root-canal-treated one.[17,18] Pulp tissue regeneration involves either delivery of autologous/allogenic stem cells into the root canals or implantation of the pulp that is grown in the laboratory using stem cells. Both these techniques will have certain advantages and limitations that need further research.[19]

A landmark study conducted by Gronthos *et al*. demonstrated both *in vitro* and *in vivo* in animals that dental pulp stem cells (DPSCs) were capable of forming ectopic dentin and associated pulp tissue.[20,21] Batouli *et al*. used an *in vivo* stem cell transplantation system to investigate differential regulation mechanisms of bone marrow stromal stem cells (BMSCs) and DPSCs. DPSCs were found to be able to generate a reparative dentin-like tissue on the surface of human dentin *in vivo*. This study provided direct evidence to suggest that osteogenesis and dentinogenesis mediated by BMSCs and DPSCs, respectively, may be regulated by distinct mechanisms, leading to the different organization of the mineralized and nonmineralized tissues.[22]

### Periodontal regeneration

Regenerating the periodontium has always been a high priority in craniofacial regenerative biology. Due to the complex structure of the periodontium (consisting of hard and soft tissues), its complete regeneration has always remained a challenge. All the current regenerative techniques such as autologous bone grafts, allografts, or alloplastic materials have limitations and cannot be used in all clinical situations. Therefore, a cell-mediated bone regeneration technique will be a viable therapeutic alternative. Kawaguchi *et al*. demonstrated that the transplantation of *ex vivo* expanded autologous MSCs can regenerate new cementum, alveolar bone, and periodontal ligament in class III periodontal defects in dogs.[23] Going a step further, periodontal ligament cells cultured *in vitro* were successfully reimplanted into periodontal defects in order to promote periodontal regeneration by Hasegawa *et al*.[24] A subsequent study by the same group reported a similar approach in humans. This study reported firm evidence that stem cells can be used to regenerate a tissue as complex as the periodontium.[25]

### Repair and regeneration of bone in craniofacial defects

Craniofacial bone grafting procedures rely on autologous bone grafting, devitalized allogenic bone grafting (using bone from bone bank), and natural/synthetic osteoconductive biomaterials. Autologous bone grafting is limited by donor site morbidity and allogenic bone is often destroyed soon. A long-term outcome using biomaterials relies on their ability to encourage local cells to completely regenerate a defect and results are often not encouraging. If stem cells can be harvested in a scaffold and transplanted into a defect to regenerate the lost tissue, it can alleviate a lot of complications associated with the traditional techniques. Abukawa *et al*. used a novel scaffold design with a new fabrication protocol to generate an autologous tissue engineered construct which was used to repair a segmental mandibular defect. The technique promoted osteogenesis and enhanced penetration of bone with blood vessels thereby accelerating tissue regeneration.[26] In a dog model, Yamada *et al*. showed that a mixture of MSCs and platelet-rich plasma improved bone implant contact and bone density in a mandibular defect.[27] The development of new scaffold fabrication technologies has facilitated a successful repair of three dimensionally complex cranial defects.[28] To further enhance the regenerative potential of MSCs, genetic engineering technologies have been utilized to extend the life of stem cells and to enhance osteogenesis.[29–31] In summary, cell-derived therapy for the repair of osseous defects has been relatively successful and numerous clinical trials in human craniofacial defects are underway.

### Whole tooth regeneration

A therapeutic option that was unthinkable a few years ago seems an achievable goal today. Even to this day, the replacement of missing teeth has limitations. Although, implants are a significant improvement over dentures and bridges, their fundamental limitation is the lack of natural structural relationship with the alveolar bone (absence of periodontal ligament). They rely on direct integration of bone on tooth surface which is indeed an unnatural relationship as compared with the natural tooth. Further, they are also associated with a lot of esthetic, functional, and surgical limitations that affect their prognosis. Ohazama *et al*. reported the reconstruction of murine teeth using cultured stem cells which when transferred into renal capsules resulted in the development of tooth structures and associated bone.[32] Nakao *et al*. recently engineered teeth ectopically and transplanted them into an anthrotopic site in a mouse jaw.[33] Sonayama *et al*. used SCAP and PDLSCs and formed a bioroot in mini pigs. SCAP and PDLSCs were seeded in a scaffold and implanted into the sockets of the lower jaw. Postchannels were precreated to leave space for postinsertion and 3 months later the bioroot was exposed and a porcelain crown was inserted. The bioroot developed, and had a natural relationship with the surrounding bone.[34]

## NATURE VERSUS NURTURE

Despite the significant advances made in medicine over the years, replacing the experienced processes perfected by nature is indeed a difficult proposition. We are yet to get a suitable blood substitute, and still rely on “blood for blood,” and “tissue for tissue.” Although there is little doubt that the best material to replace a tooth or tooth-related tissue is the tooth tissue itself, the question that lies before us is “Can we do it in a way that is predictable and is it practical/clinically feasible?” For any new therapy to be accepted, it should either be more effective than or as effective as the existing therapy with added advantages in terms of technique being simpler, more predictable, long-lasting, time/cost-effective, etc. If the above goal has to be achieved and if laboratory research has to be translated into clinical practice, there are certain barriers that have to be first understood and later overcome.

### Barriers to overcome

To regenerate tooth/tooth-related tissues, the prerequisites are:

- Stem cells
- Signaling molecules – to induce differentiation into the required tissue type, i.e., into osteoblasts, odontoblasts, cementoblasts, fibroblasts, etc.
- Scaffold material to support and harvest cellular proliferation.

### Stem cell source

Stem cells obtained from any adult tissue are less, about 1–4%.[35] Further, their isolation, expansion, and storage is a very technique-sensitive procedure. When banked, how long can they be safely stored retaining the original stemness is questionable. MSCs are considered immunomodulatory as they lack MHC type II (major histocompatibility complex) antigen and therefore do not provoke immune reactions. However, foolproof evidence for their immune suppression characteristics needs to be established.[36] As of now, autologous stem cells are ideally suited for a patient as there is no risk of immune rejection, the process is least expensive, and avoids legal and ethical concerns. The cells need to be procured from an autologous source, and isolated and expanded in number before they can be used and hence the process is time consuming.

The use of pre-existing allogenic cell lines and cell organ culture removes the problem of harvesting cells from the patients themselves and saves considerable time.[37] One of the serious questions that have remained unanswered is “Are allogenic stem cells safe in terms of immune suppression and pathogen transmission?”[38] However, there are many *in vivo* studies which support that they is immunologically safe.[39,40]

### Signaling molecules

There are many growth factors that play a role in tissue regeneration. Growth factors are proteins that bind to receptors on the cell and induce cellular proliferation and/or differentiation.[41] Dentin contains many proteins that are capable of stimulating tissue responses. The demineralization of dentin tissue due to caries can itself lead to the release of growth factors.[42] It is also suggested that the therapeutic effect of calcium hydroxide may be because of the extraction of growth factors from dentin matrix.[43] Once released, these growth factors may play a role in signaling many of the events of tertiary dentinogenesis, a response of pulp-dentin repair.[44,45] Two important families of growth factors that play a vital role are the transforming growth factor-beta (TGF-β) family[46,47] and bone morphogenic proteins (BMPs).[48–52] Although we are aware of the role played by these signaling molecules, we are yet to have clarity on how these signals can be spatially distributed in a right combination, time, and sequence which indeed is crucial to obtain predictable results.

### Scaffold

The scaffolds used for regeneration should be biodegradable and the rate of degradation should coincide with the rate of tissue formation. They should be highly porous to facilitate cell seeding and diffusion of nutrients. While cells are fabricating their own natural matrix structure around themselves, the scaffolds should provide structural integrity and then eventually breakdown leaving the newly formed tissue. During this entire process, they should maintain important characteristics of stem cells and yet allow for appropriate differentiation of their progeny.[53,54]

#### Scaffold materials

Several scaffolds have been tried which include both natural and synthetic materials. Natural materials like collagen, alginate, agarose,[55] chitosan, and glycoseaminoglycans (GAGs)[56,57] have been tried. Synthetic materials have been more extensively investigated which include hydroxyapetite/tricalcium phosphate, and polymers like polylactic acid, polyglycolic acid, and polycaprolactone. Synthetic polymers are found to be more conductive and show less contraction as compared to collagen.[58,59]

#### Scaffold designs and delivery

To regenerate bone and other body parts, it is preferable to have rigid tissue-engineered scaffold structures as they provide an excellent physical support to the cells.[60] However, in the root canal system it is preferable to have a soft three-dimensional scaffold matrix such as polymer hydrogel which can be noninvasive and easily delivered by injecting into the root canal systems.[61,62] Hydrogels are in the early stage of research and yet to be proven *in vivo*. Research is on in making them photopolymerizable to form rigid structures once they are implanted into any tissue site.[63] Another problem in tissue regeneration is vascularization of the area. Enhanced vascularization is needed in order to support the vitality of the implanted cells in the scaffold. Efforts are on in developing a scaffold system that promotes angiogenesis of the vascular network by impregnating the scaffold with growth factors like vascular endothelial growth factor (VEGF) and/or platelet-derived growth factor or further with an addition of endothelial cells. These techniques are particularly important for pulp tissue engineering as the blood supply is only from the apical end.[64–69]

## CONCLUSION

Stem cells derived from all sources hold immense medical promises. Stem cell therapies have virtually unlimited medical and dental applications. While there are several barriers that need to be broken down before this novel therapy can be translated from lab to clinics, it is certain that the future is going to be exciting for all of us. We have moved on from the surgical model of care to the medical model and are likely to move onto the biological model of care. The need of the hour is high-quality research coupled with collaboration between basic scientists and the clinicians. A team effort engaging the expertise of the molecular biologists, immunologists, biomaterial scientists, cell biologists, matrix biologists, and practicing dental surgeons is crucial in attaining the desired goal. Stem cell therapy is no longer science fiction. Recent developments in the technique of stem cell isolation and expansion together with advances in growth factor biology and biodegradable polymer constructs have set a stage for successful tissue engineering of tooth/tooth-related tissues. Stem cell therapy has brought in a lot of optimistic hope amongst researchers, doctors, and not to forget the patients who are the chief beneficiary of this innovation. Stem cells regenerate hope and not all that is happening in research is hype.

Remember – “Hope is a prerequisite for any successful scientific innovation.” And one day, very soon, stem cells will be introduced as an alternate/adjuvant in medicine and dentistry.
